# The intestinal virome in children with cystic fibrosis differs from healthy controls

**DOI:** 10.1371/journal.pone.0233557

**Published:** 2020-05-22

**Authors:** Michael J. Coffey, Ivan Low, Sacha Stelzer-Braid, Bernd Wemheuer, Millie Garg, Torsten Thomas, Adam Jaffe, William D. Rawlinson, Chee Y. Ooi

**Affiliations:** 1 School of Women's and Children's Health, University of New South Wales, Sydney, New South Wales, Australia; 2 School of Biotechnology and Biomolecular Sciences, University of New South Wales, Sydney, New South Wales, Australia; 3 Virology Research Laboratory, Prince of Wales Hospital, Sydney, New South Wales, Australia; 4 School of Medical Sciences, University of New South Wales, Sydney, New South Wales, Australia; 5 School of Biological, Earth and Environmental Sciences, Centre for Marine Science and Innovation, University of New South Wales, Sydney New South Wales, Australia; 6 Department of Respiratory, Sydney Children's Hospital Randwick, Sydney, New South Wales, Australia; 7 Molecular and Integrative Cystic Fibrosis (miCF) Research Centre, Sydney, New South Wales, Australia; 8 Department of Gastroenterology, Sydney Children's Hospital Randwick, Sydney, New South Wales, Australia; Hospital Universitario Ramon y Cajal, SPAIN

## Abstract

Intestinal bacterial dysbiosis is evident in children with cystic fibrosis (CF) and intestinal viruses may be contributory, given their influence on bacterial species diversity and biochemical cycles. We performed a prospective, case-control study on children with CF and age and gender matched healthy controls (HC), to investigate the composition and function of intestinal viral communities. Stool samples were enriched for viral DNA and RNA by viral extraction, random amplification and purification before sequencing (Illumina MiSeq). Taxonomic assignment of viruses was performed using Vipie. Functional annotation was performed using Virsorter. Inflammation was measured by calprotectin and M2-pyruvate kinase (M2-PK). Eight CF and eight HC subjects were included (50% male, mean age 6.9 ± 3.0 and 6.4 ± 5.3 years, respectively, p = 0.8). All CF subjects were pancreatic insufficient. Regarding the intestinal virome, no difference in Shannon index between CF and HC was identified. Taxonomy-based beta-diversity (presence-absence Bray-Curtis dissimilarity) was significantly different between CF and HC (R^2^ = 0.12, p = 0.001). *Myoviridae*, *Faecalibacterium* phage FP Taranis and unclassified *Gokushovirinae* were significantly decreased in CF compared with HC (q<0.05). In children with CF (compared to HC), the relative abundance of genes annotated to (i) a peptidoglycan-binding domain of the peptidoglycan hydrolases (COG3409) was significantly increased (q<0.05) and (ii) capsid protein (F protein) (PF02305.16) was significantly decreased (q<0.05). *Picornavirales*, *Picornaviridae*, and *Enterovirus* were found to positively correlate with weight and BMI (r = 0.84, q = 0.01). Single-stranded DNA viruses negatively correlated with M2-PK (r = -0.86, q = 0.048). Children with CF have an altered intestinal virome compared to well-matched HC, with both taxonomic and predicted functional changes. Further exploration of Faecalibacterium phages, *Gokushovirinae* and phage lysins are warranted. Intestinal viruses and their functions may have important clinical implications for intestinal inflammation and growth in children with CF, potentially providing novel therapeutic targets.

## Introduction

Dysfunction of the cystic fibrosis (CF) transmembrane conductance regulator (CFTR) results in an altered intestinal milieu with proposed mechanisms including: (i) reduced bicarbonate secretion and low intestinal pH, (ii) thick and inspissated mucus, (iii) a lack of endogenous pancreatic enzymes, (iv) delayed intestinal transit and (v) possibly impaired innate immunity [1, 2]. These mechanisms along with treatment and diet regimens [3] likely result in intestinal bacterial dysbiosis and inflammation, which have repeatedly been reported in CF [4–12]. Intestinal inflammation in CF may have significant clinical relevance due to its link with growth and nutrition [9, 13, 14]. In children with CF compared to healthy controls (HC), intestinal bacterial profiles are often characterised by an increased abundance of Proteobacteria (*Escherichia coli*, *Shigella*, *Enterobacter*) and a decreased abundance of Bacteroidetes (*Bacteroides*, *Alistipes*) and Firmicutes (*Faecalibacterium prausnitzii*) [4, 6, 8, 12]. It has been suggested that in children with CF, enteric fat abundance (increased as a result of diet regimens and a lack of endogenous pancreatic enzymes) selects for pro-inflammatory gut microbiota [6]. Additionally, the faecal microbiota of people with CF may have a higher prevalence of amoxicillin resistance [15].

Intestinal viruses also have the potential to contribute to bacterial dysbiosis and intestinal inflammation, given their influence on bacterial species diversity and biochemical cycles. To the best of our knowledge, the intestinal virome has yet to be explored in CF, both in terms of characterisation and its potential contribution to microbiota, inflammation and CF pathogenesis.

Human faecal viromes typically consist of eukaryote-infecting viruses, and bacteriophages (viruses of bacterial hosts). Predominant bacteriophages in the gut include double-strand DNA (dsDNA) *Caudovirales* and single-strand DNA (ssDNA) *Microviridae* [16–19]. The community of intestinal bacteriophages show substantial inter-individual variability, but appear temporally stable within a single individual [16, 17]. Bacteriophages can influence bacterial populations via host lysis and horizontal gene transfer, as well as playing an indirect role in regulating immune function and inflammation [19–22]. Specifically, inflammatory bowel disease studies have shown changes in the intestinal virome (compared to HC) are likely due to changes in lytic phage populations, which lyse bacterial hosts, resulting in release of pathogen-associated molecular patterns and antigens that cause inflammation [19, 23].

In recent years there has been a focus on the role of viruses in CF lung disease [24]. In CF airways, environmental stress and frequent antibiotic treatment has been suggested to enhance bacteriophage mobility and promote bacteriophage-mediated spread of antibiotic resistance genes [25, 26]. The respiratory viromes of adults with CF have previously been explored in several small studies using a metagenomics approach, [27–30] however only Willner et al. (2009) [27] included healthy controls in their study. To the best of our knowledge, the intestinal virome in CF has not been explored, particularly in comparison to matched-healthy controls.

The aim of this study was to investigate the composition and function of viral communities inhabiting the intestines of children with CF. We hypothesized that the composition and functional capacity of viral communities in the intestines of children with CF are different when compared to HC. Furthermore, we hypothesized that these alterations have clinical implications in children with CF.

## Results

### Population characteristics

There were eight children with CF and eight paired HC matched for age and gender included in this study. The mean age of CF and HC subjects was 6.9 ± 3.0 and 6.4 ± 3.2 years, respectively (p = 0.8), with a range of 3 to 12 years for both cohorts. Each group consisted of four males (50%). All CF children were pancreatic insufficient (PI) and five were homozygous for the F508del mutation in the *CFTR* gene (a disease-causing CF mutation) (63%), whilst the remaining three were heterozygous for the F508del mutation and a second disease-causing mutation (37%). No CF participants were on CFTR modulator therapy. Clinical characteristics are presented in Table 1.

**Table 1:** Clinical characteristics.

ID	Gender	Group	Pancreas	Age (years)	Genotype	Calprotectin(mg/kg)	M2-PK (U/ml)
CF1	M	CF	PI	3.0	F508del / F508del	204.7	142.5
HC1	M	HC	HC	3.0	- / -	-	-
CF2	M	CF	PI	3.1	F508del / F508del	56.8	35.0
HC2	M	HC	HC	3.2	- / -	17.6	< 1
CF3	F	CF	PI	5.8	F508del / F508del	171.4	6.1
HC3	F	HC	HC	3.1	- / -	97.3	< 1
CF4	F	CF	PI	6.5	F508del / G551D	45.2	7.4
HC4	F	HC	HC	5.8	- / -	30.5	< 1
CF5	M	CF	PI	8.2	F508del / F508del	51.7	10.1
HC5	M	HC	HC	8.6	- / -	-	-
CF6	M	CF	PI	8.4	F508del / 394delTT	141.1	5.2
HC6	M	HC	HC	11.7	- / -	< 19.5	3.0
CF7	F	CF	PI	8.8	F508del / F508del	< 19.5	6.4
HC7	F	HC	HC	7.6	- / -	-	-
CF8	F	CF	PI	11.8	F508del / G551D	149.2	-
HC8	F	HC	HC	8.5	- / -	< 19.5	< 1

Demographics and fecal inflammatory marker results of participants. No CF participants were on CFTR modulator therapy. M2-PK, M2-pyruvate kinase; PI, pancreatic insufficient; -, not tested or data not available.

### Virome characteristics

Viral community structures were successfully assessed using Illumina MiSeq sequencing of a viral DNA and RNA enrichment of stool samples. Sequencing data were processed using the Vipie pipeline and contained an average of 3,909,006 ± 1,283,246 reads per sample, which after quality filtering and de novo assembly resulted in a median (IQR; range) of 863 (625–2946; 227–7737) contigs per sample. On average, samples contained 568,964 ± 201,042 reads that were mapped to viruses (75.6% ± 27.5%), bacterial ribosomes (23.7% ± 27.2%) and humans (0.63% ± 0.88%) (S3 Data). Bacterial contamination rates (based on reads mapping to bacterial ribosomes) ranged from 0.1% to 82.2% and both bacterial and human reads were filtered out from the analysis. Rarefaction curves of viral taxa suggest the number of samples included in this study did not reach virome saturation, suggesting that a larger number of samples per cohort would be needed to capture the entire human virome (S1A and S1B Fig). However, saturation of rarefaction curves for individual samples was achieved, suggesting that all viruses in a given sample were recovered (S1C and S1D Fig).

### Alpha diversity of virome

**Fig 1:**
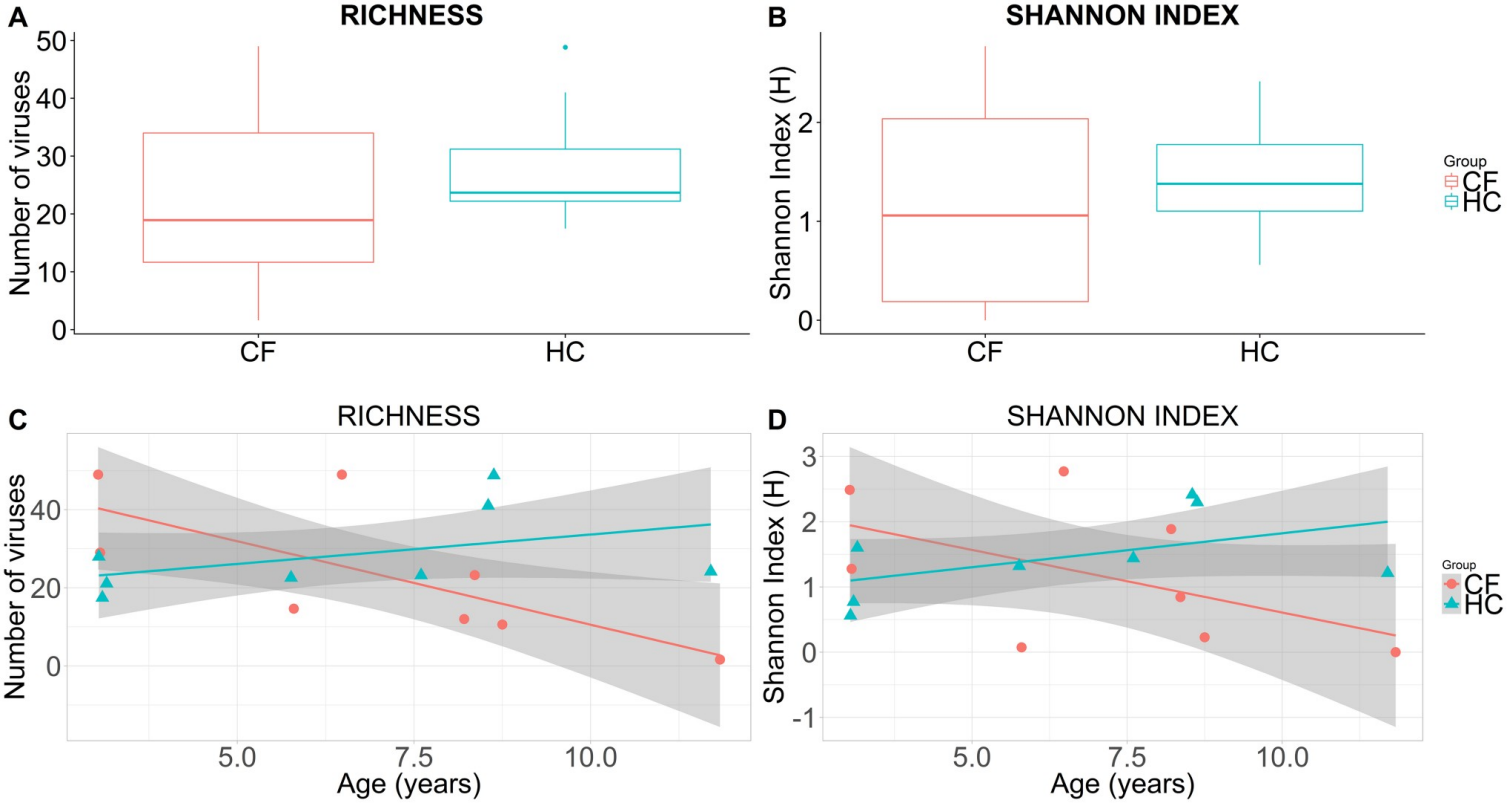
Boxplots of sample richness (number of unique viruses) (A) and Shannon index (B) in CF and HC cohorts. Scatterplots of sample richness (number of unique viruses) (C) and Shannon index (D) against age in CF and HC cohorts. Cohort mean and 95% confidence intervals are constructed from generalised linear models and presented as lines and shaded regions, respectively (C, D).

Regarding the virome alpha diversity, children with CF and HC had no significant difference in richness (number of unique viruses) (mean difference (95% CI) of -4.7 (-24.9 to 15.6), p = 0.6) and Shannon index (mean difference (95% CI) of -0.3 (-1.4 to 0.9), p = 0.6) (Fig 1A and 1B). Controlling for age, there was also no difference between children with CF and HC for richness (estimate (SE) -4.1 (7.4), p = 0.6) and Shannon index (estimate (SE) -0.2 (0.5), p = 0.6). However, with advancing age, alpha diversity indices (richness and Shannon index) decreased in CF and increased in HC cohorts (Fig 1C and 1D). The intercepts for the two generalised linear models that fit the trends were significantly different (richness model p = 0.008; Shannon index model p = 0.045) suggesting the effect of age should be considered.

### Beta diversity of virome

**Fig 2:**
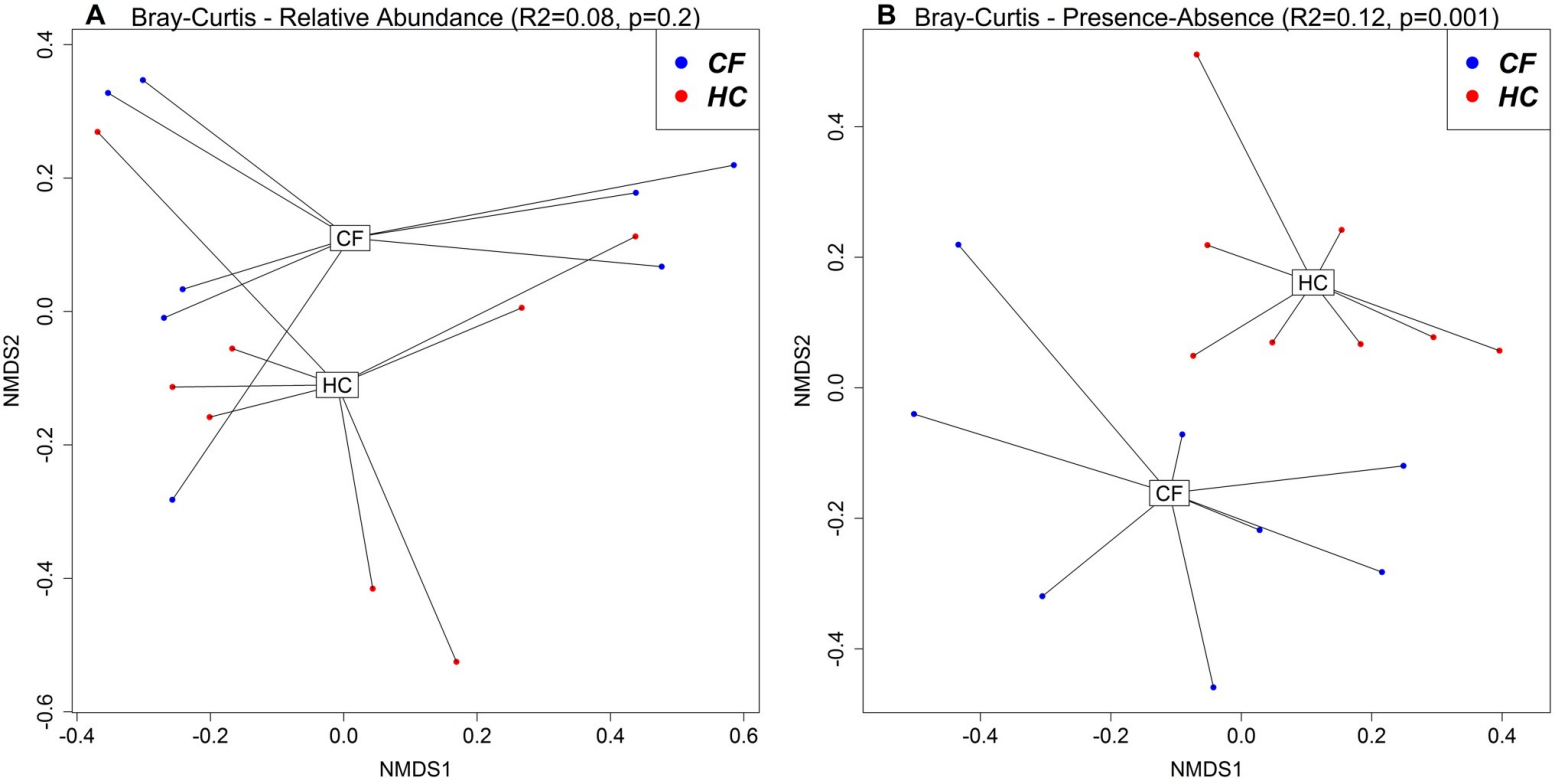
NMDS plots based on Bray-Curtis dissimilarities using relative abundance (A) and presence-absence (B) data between CF and HC cohorts.

Visualization of taxonomy-based beta diversity (Fig 2) showed a significant difference in viral communities between CF and HC cohorts based on presence-absence data using Bray-Curtis dissimilarity (BC) (R^2^ = 0.12, p = 0.001), but not on relative abundance data using BC (R^2^ = 0.08, p = 0.2). PERMANOVA showed no significant difference in viral communities between males and females (relative abundance BC R^2^ = 0.09, p = 0.09; presence-absence BC R^2^ = 0.07, p = 0.2). PERMANOVA showed a significant difference in viral communities across ages (relative abundance BC R^2^ = 0.11, p = 0.02; presence-absence BC R^2^ = 0.09, p = 0.04).

### Differences in viral communities between CF and HC populations

**Fig 3:**
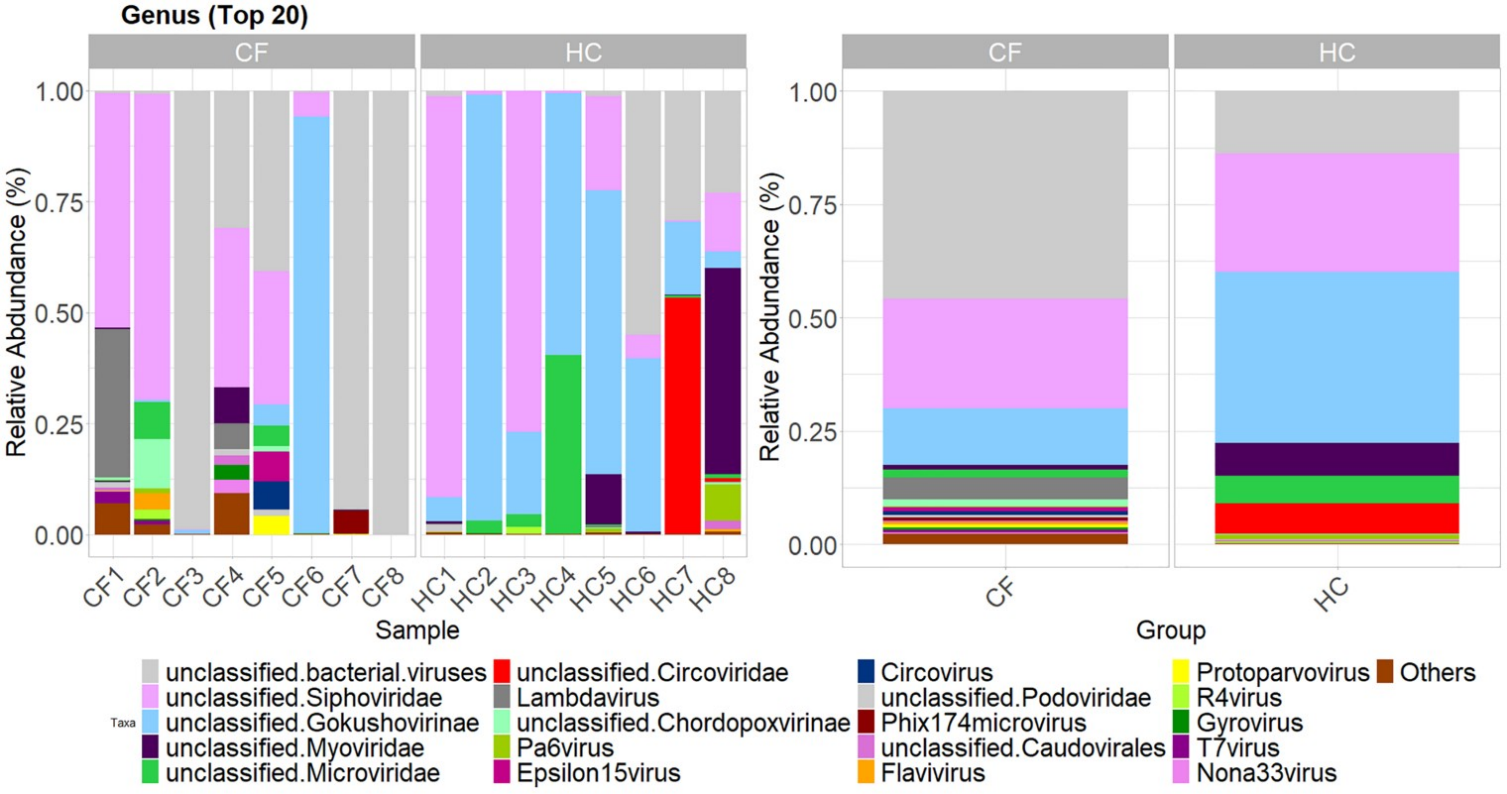
Relative abundance of top 20 most abundant viral genera for CF and HC subjects. Samples ordered in increasing age (from left to right).

The relative abundance of all viruses at each of the taxonomic ranks in CF and HC subjects are presented in S2A–S2E Fig. Relative abundances of the top 20 most abundant viral genera in CF and HC are presented in Fig 3. The relative abundance of *Myoviridae* (family) and one of its species, *Faecalibacterium* phage FP Taranis were significantly decreased in children with CF compared with HC (q<0.05) (S3A and S3C Fig). Unclassified *Gokushovirinae* was also significantly decreased in children with CF compared with HC (q<0.05) (S3B Fig). Unclassified *Gokushovirinae* consisted of two species of *Gokushovirus* (WZ-2015a and *human gut gokushovirus* isolate SH).

Porcine circovirus (PCV) and porcine parvovirus (PPV) are known to be in porcine-derived pancreatic enzyme replacement therapy (PERT) products prescribed to patients with CF [31, 32]. We identified PCV type 2 (PCV2) and PPV in 4/8 (50%) and 3/8 (38%) of CF subjects respectively, and not in any HC subjects.

### Functional annotations

**Fig 4:**
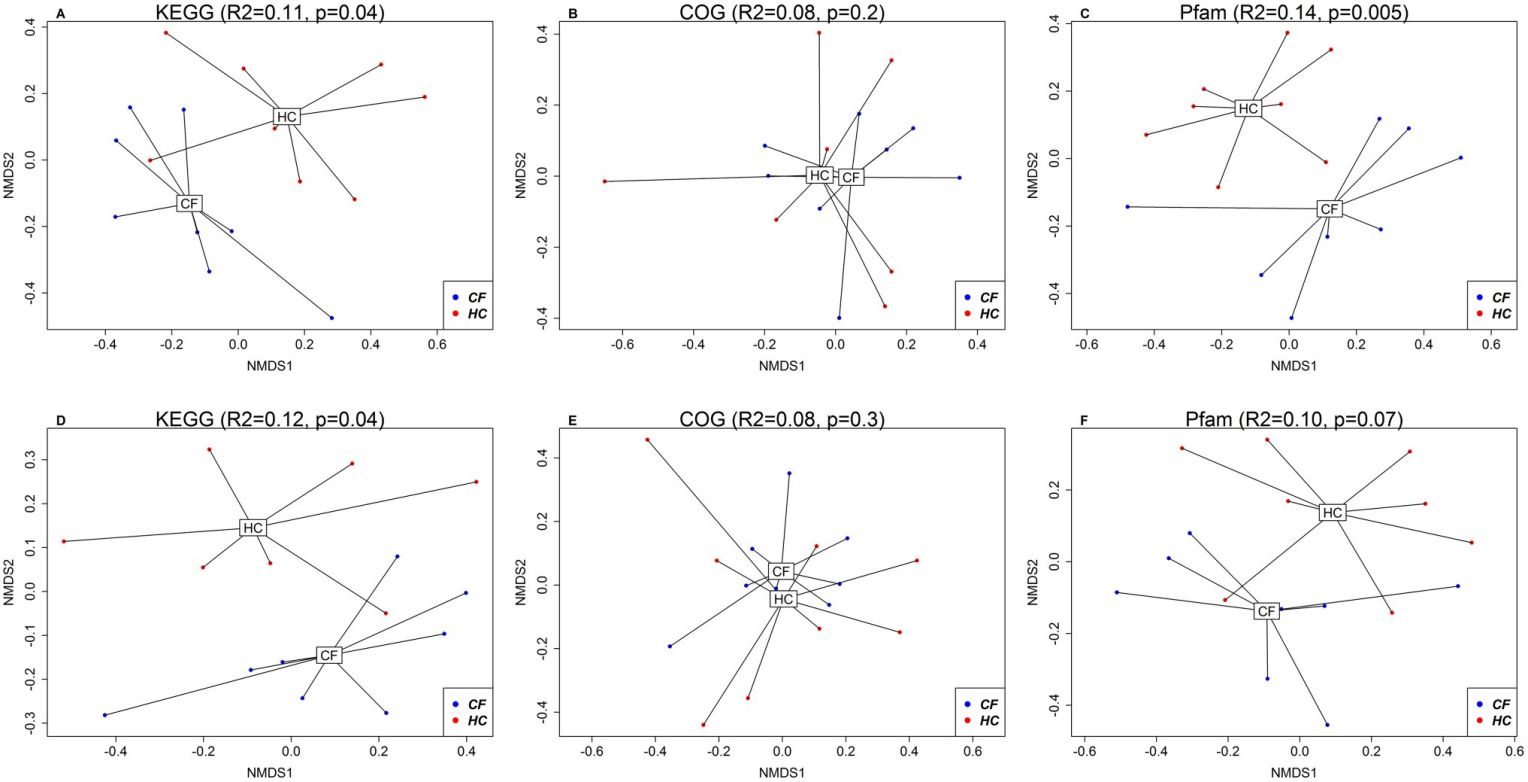
NMDS plots based on Bray-Curtis dissimilarities using relative abundance (A-C; top row) and presence-absence (D-F; bottom row) data on functional annotation (using KEGG, COG and Pfam databases) between CF and HC cohorts.

The functional annotations were performed using Virsorter [33] to search against the Kyoto Encyclopedia of Genes and Genomes (KEGG) [34], Clusters of Orthologous Groups (COG) [35] and Pfam [36] databases. On average, 225 (138) predictions were identified per sample. Predictions were mapped to: (i) 71 unique KEGG terms (4.8% predictions mapped); (ii) 146 unique COG terms (10.4% predictions mapped); and (iii) 223 unique Pfam terms (16.6% predictions mapped). Visualization of beta diversity based on the functional annotation (Fig 4) showed a significant difference in KEGG terms between CF and HC cohorts based on relative abundance data using BC dissimilarity (R^2^ = 0.11, p = 0.04) and presence-absence data using BC dissimilarity (R^2^ = 0.12, p = 0.04). There was no significant difference in COG terms between CF and HC cohorts based on relative abundance BC (R^2^ = 0.08, p = 0.2) and presence-absence BC (R^2^ = 0.08, p = 0.3). There was a significant difference in Pfam terms between CF and HC cohorts based on relative abundance BC (R^2^ = 0.14, p = 0.005), but not on presence-absence BC (R^2^ = 0.10, p = 0.07).

The relative abundance of peptidoglycan-binding (PGRP) domain of the peptidoglycan hydrolases (COG3409 [COG]) was significantly elevated in children with CF compared with HC (q<0.05) (S4A Fig). The relative abundance of capsid protein (F protein) (PF02305.16 [Pfam]) was significantly decreased in children with CF compared with HC (q<0.05) (S4B Fig). There was no significant difference in the relative abundance of any KEGG terms between CF and HC cohorts (q<0.05), however zinc D-Ala-D-Ala carboxypeptidase (K08640 [KEGG]) came close to significance (q<0.1) (S4C Fig).

### Age-related changes in the intestinal virome

Exploring the effect of age, linear models of the differentially abundant viruses and protein sequences were constructed and presented in S5 Fig. Comparing children with CF and HC whilst controlling for age, the relative abundance of: (i) *Myoviridae* was significantly decreased with age in CF (estimate (SE) -1.1×10^−4^ (3.7×10^−5^), p = 0.01); (ii) unclassified *Gokushovirinae* was not significantly different (estimate (SE) -0.02 (0.01), p = 0.2); (iii) *Faecalibacterium* phage FP Taranis was significantly decreased in CF (estimate (SE) -4.5×10^−5^ (1.6×10^−5^), p = 0.02); (iv) a peptidoglycan-binding (PGRP) domain of peptidoglycan hydrolases (COG3409) was significantly increased in CF (estimate (SE) 2.0×10^−3^ (4.5×10^−4^), p = 0.0006); (v) capsid protein (F protein) (PF02305.16) was significantly decreased in CF (estimate (SE) -0.008 (0.002), p = 0.003); and (vi) zinc D-Ala-D-Ala carboxypeptidase (K08640) was significantly decreased in CF (estimate (SE) -0.005 (0.001), p = 0.009) and significantly increased with age in HC (estimate (SE) 0.0007 (0.0003), p = 0.02);

### Associations with anthropometrics and inflammatory biomarkers in children with CF

In children with CF, there were no significant correlations between alpha diversity indices (richness and Shannon diversity index) and anthropometric (height, weight and body mass index (BMI)) z-scores or intestinal inflammatory makers (calprotectin and M2-PK). Correlations between the relative abundances of viruses with anthropometric z-scores and intestinal inflammatory makers in children with CF are presented in S1 Table. *Picornavirales*, *Picornaviridae* and *Enterovirus* positively correlated with weight and BMI (r = 0.84, q = 0.01). *Anelloviridae* also positively correlated with weight and BMI (r = 0.79, q = 0.03). *Parvoviridae* and *Protoparvovirus* negatively correlated with weight (r = -0.87, q = 0.03).

Regarding inflammatory markers, children with CF had significantly elevated median (IQR) faecal calprotectin levels compared to HC (98.9 mg/kg (50.1–104.9) vs. 19.5 mg/kg (19.5–30.5), respectively, p = 0.047; lower limit of detection was 19.5 mg/kg) (S6A Fig). Median (IQR) faecal M2-PK levels were also significantly elevated in CF compared with HC (7.4 U/ml (6.3–22.6) vs. 1.0 U/ml (1.0–1.0), respectively, p = 0.005; lower limit of detection was 1.0 U/ml) (S6B Fig). *Podoviridae* positively correlated with M2-PK (r = 0.85, q = 0.03). Single-stranded DNA viruses and unclassified ssDNA viruses (order) negatively correlated with M2-PK (r = -0.86, q = 0.048).

Correlations between the relative abundances of KEGG, COG and Pfam terms with anthropometric z-scores and intestinal inflammatory makers in children with CF are presented in S2 Table. N-acetylmuramoyl-L-alanine amidase (K01447) positively correlated with height (r = 0.85, q = 0.02). No correlations with q<0.05 were identified between COG or Pfam terms and: (i) anthropometric z-scores, or (ii) intestinal inflammatory markers.

## Discussion

We have demonstrated the intestines of children with CF exhibit an altered intestinal virome, which includes both taxonomic and functional changes, when compared to matched healthy controls. To our knowledge, this is the first study to explore the intestinal virome in children with CF using a metagenomics approach on an enriched virome preparation. We observed high inter-individual variability in intestinal viruses and distinct clustering (based on presence-absence data for the BC analysis) between CF and HC cohorts. The results of this study suggest that the virome has a potential role in intestinal inflammation and growth impairment in children with CF. Whether this is a direct influence or indirectly through bacterial population changes is unknown and will require further investigation. This could be one explanation for why therapies directed at specifically modulating microbiota (e.g. probiotics [37]) may not be sufficient. A holistic approach that incorporates other microbes (i.e. viruses) may be necessary to restore intestinal homeostasis in children with CF.

The intestinal inflammation present in children with CF is clinically relevant given its negative correlation with growth (9). We have identified distinct mechanisms which may promote intestinal inflammation in children with CF, including: (i) excess production of bacteriophage endolysins (e.g. peptidoglycan-binding (PGRP) domain of peptidoglycan hydrolases) which have potential pro inflammatory effects [19, 36], (ii) under expression of viruses which lyse obligate intra-cellular parasitic bacteria (e.g. *Gokushovirinae*) [38] and bacteriophages associated with beneficial bacteria, *Faecalibacterium prausnitzii* (e.g. *Faecalibacterium* phage FP Taranis) [39, 40], and (iii) over expression of bacteriophages associated with potentially detrimental Proteobacteria (e.g. *Podoviridae*) [41]. Additionally, we identified several viruses which positively (e.g. *Anelloviridae* and *Enterovirus*) and negatively (e.g. *Protoparvovirus*) correlated with growth measures in children with CF. Functional annotation of intestinal viruses revealed distinct clusters between CF and HC when referencing the KEGG (Fig 4A and 4D) and Pfam (based on relative abundance BC) databases (Fig 4C). A peptidoglycan-binding (PGRP) domain of peptidoglycan hydrolases (COG3409) was significantly elevated in children with CF compared to HC. These endolysins are synthesised by bacteriophages at the end of their lytic cycle and result in the degradation of their host’s peptidoglycan-based cell wall to allow for the release of viral progeny [36]. This finding may provide one hypothesis for the reduced bacterial richness and Shannon index previously reported in CF children [4, 6]. Furthermore, lysis of bacteria may release nucleic acids, proteins and lipids that serve as inflammatory response triggers, inducing cellular infiltration, cytokine production and even tissue damage [19], a possible contributory factor to the elevated M2-PK in children with CF.

Consistent with current literature [16–19], intestinal viruses were predominantly bacteriophages, namely dsDNA *Caudovirales* and ssDNA *Microviridae*. *Gokushovirinae* were significantly decreased in children with CF. *Gokushovirinae* are generally considered lytic viruses and typically infect obligate intra-cellular parasitic bacteria, e.g. *Chlamydia* and *Spiroplasma* [38]. Furthermore, *Gokushovirinae* peptidases resemble peptidases from Firmicutes (e.g. *Faecalibacterium*) and Proteobacteria (e.g. *Ahrensia*, *Providencia*) suggesting a horizontal acquisition of genes during a co-infection or infection of the prophage-bearing host cell [42]. The importance of ssDNA viruses in CF intestinal dysbiosis, which were predominantly *Gokushovirinae* in our cohort, are further emphasised by the negative correlation with M2-PK. M2-PK is expressed by rapidly proliferating intestinal cells and elevated in the setting of intestinal inflammation and colorectal cancer [43], however the mechanism for a negative correlation with ssDNA viruses is unclear. The decreased relative abundance of *Gokushovirinae* in our CF cohort is also consistent with the reduction of capsid protein (F protein), which is a major capsid component of ssDNA bacteriophages [36].

The relevance of intestinal viruses and inflammation is highlighted by *Faecalibacterium* phage FP Taranis, which was significantly decreased in children with CF. The bacterial host for this phage is *F*. *prausnitzii* [39], which has previously been reported to be decreased in children and adolescence with CF [44], as well as in other inflammatory conditions including inflammatory bowel disease and psoriasis [45]. *F*. *prausnitzii* is considered a beneficial bacteria as it is an important butyrate producer with anti-inflammatory activity through the production of MAM protein [40]. Therefore, reduced *Faecalibacterium* phage FP Taranis may be reflective of reduced *F*. *prausnitzii* and reduced anti-inflammatory capacity i.e. resulting in elevated inflammatory biomarkers (calprotectin and M2-PK) in children with CF. Additionally, *Podoviridae* (a family of dsDNA viruses) positively correlated with M2-PK and several phage from this family are associated with potentially detrimental Proteobacteria (*Enterobacteria*, *Escherichia*, *Pseudomonas* and *Salmonella* Phage) [41].

The associations between intestinal viruses and anthropometrics in children with CF require further exploration as the potential mechanisms are unclear. We identified that in children with CF, the family *Anelloviridae* and the genus *Enterovirus* positively correlated with weight and BMI z-scores (S1 Table). A phage lysin, N-acetylmuramoyl-L-alanine amidase (K01447) also positively correlated with height z-scores in children with CF (S2 Table). *Protoparvovirus* negatively correlated with weight z-scores and the only identified species of this genus was PPV. Porcine parvovirus is currently not thought to infect humans [46] and the negative correlation of *Protoparvovirus* with weight is more than likely reflective of an increase in PERT dosing to improve weight gain in our CF clinic. The presence of the ssDNA viruses PPV and PCV2 only in CF subjects may have unintentionally provided quality assurance of our workflow, given that they are both known to be in porcine-derived PERT products prescribed to patients with CF [31, 32]. *Porcine circovirus* 2 is ubiquitous among swine populations and not thought to infect immunocompetent humans [47].

The trends in alpha diversity indices between CF and HC cohorts with advancing age appeared divergent (Fig 1C and 1D) and there was a significant difference in viral communities across ages in all children. Additionally, children with CF clustered separately from HC (Fig 2B), despite the high degree of inter-individual variability (Fig 2A and 2B). These findings highlight the need to further explore and/or control for age in future studies.

Our study is limited by the developing nature of virus reference databases. Unknown viruses may influence our estimates of alpha and beta diversity. Our sequencing effort appeared adequate for individual samples (saturation of rarefaction curves S1C and S1D Fig), and similar to Shkoporov et al. (2019) [48], with 75% of reads mapped to viruses. A larger sample size is required for future studies to account for the high degree of inter-individual variability in the intestinal virobiota, to get a complete picture of the virome of CF patients, and minimise the influence of outliers (which is controlled for using the ANCOM analysis [49]). Although the percentage of predictions mapped to functional terms is small, a recent review of human gut virome literature identified 18 studies, none of which performed a functional annotation [50]. Bacterial contamination rates in our sequencing data were quite varied and could require optimisation, however the level is on par with other current studies [27, 28]. Bacterial community data (i.e. 16S rRNA sequencing data) was only available for two CF and five HC participants, therefore we were unable to assess the relationships between intestinal bacteria and viruses. Paired 16S rRNA and viral sequencing data would likely provide important insights into bacteriophage-host relationships. Given our study only investigated children aged 3 to 12 years, further studies on infants, young children and adolescents are warranted, particularly in light of the age-related differences. Additionally, potential confounding factors, including antibiotic usage and altered dietary regimens between CF and HC were not controlled for with this study. Future longitudinal studies across a wider age range with a multi-omics approach will likely provide greater insights including phage-host associations, patient immune response effects and potentially unravel therapeutic targets in CF gastrointestinal disease. Mechanisms promoting intestinal inflammation in children with CF also warrant further investigation, particularly the excess production of bacteriophage endolysins and their downstream effects. Future in-vitro studies using CF-intestinal epithelial cell lines or patient derived models with intestinal organoids should be considered for future research.

In conclusion, there exists an altered intestinal virome in children with CF compared with HC, which provides novel insights into paediatric CF gastrointestinal disease. *Faecalibacterium* phage, Gokushovirinae and phage lysins are considerations for future areas of research, specifically in the context of intestinal inflammation in CF. A high degree of inter-individual variation in the intestinal virome, which changes across age, suggests that larger, longitudinal studies are warranted. Intestinal viruses and their functions may have important clinical implications for intestinal inflammation and growth in children with CF, potentially providing novel therapeutic targets.

## Materials and methods

### Study population

We performed a prospective, cross-sectional, case-control study in children with CF and HC. The subjects and samples for this analysis were collected as part of three prior studies evaluating the progression of intestinal microbiota and inflammation in children with CF [4, 7, 11]. Children with CF and HC were prospectively recruited from the outpatient clinics (CF and Orthopaedic/Plastics clinics respectively) at Sydney Children’s Hospital Randwick, Australia. Children with CF were matched to HC for gender and age (as closely as possible). We included children: (i) aged 0 to 18 years; (ii) with CF diagnosed according to the United States Cystic Fibrosis Foundation consensus criteria [51]; (iii) exocrine pancreatic insufficiency based on the 72-hour faecal fat and/or faecal elastase-1 [52, 53]; (iv) as healthy controls if they did not have CF or any gastrointestinal disease. We excluded children: (i) requiring antibiotic therapy for a pulmonary exacerbation [54] or intravenous antibiotics in the preceding four weeks before sampling; (ii) with gastroenteritis, on oral corticosteroids, probiotics and/or non-steroidal anti-inflammatory drugs in the preceding two weeks before sampling. We did not exclude children with CF on prophylactic oral or inhaled antibiotic therapy.

This study was approved by the South Eastern Sydney Area Health Service, Human Research Ethics Committee, Sydney Australia (HREC/10/240). Written informed consent was obtained from each subject or caregiver(s) and the study was carried out in accordance with the approved guidelines.

### Sample and data collection

Stool samples were collected to provide a non-invasive representation of the intestinal viral communities and environment. Specifically, stool samples reflect the luminal contents of the large intestine, however for the purpose of this manuscript, will be referred to as a representation of the intestine. A single stool sample from each subject was collected and stored immediately at –80°C, or stored at –20°C (home freezer) until transport to the laboratory for storage at –80°C. Thawing of the sample during transport did not occur. At the time of sample collection, demographic and anthropometric data (height, weight and BMI z-scores) was recorded.

### Virome pipeline

Sample preparation to enrich for faecal viruses followed an adjusted NetoVIR (Novel Enrichment Technique Of VIRomes) protocol [55]. Details are provided in S1 Methods. Briefly, sample processing involved: (i) stool being suspended in sterile phosphate-buffered solution, homogenised and centrifuged at 17,000g (ii) supernatant being filtered through a 0.8 μm PES centrifugal filter; (iii) filtered supernatant being treated with nuclease buffer and micrococcal nuclease; (iv) viral nucleic acids being extracted using the QIAamp Viral RNA Mini Kit (Qiagen) according to manufacturer’s instructions for the extraction of viral RNA and DNA, with the exception of centrifugation speed lowered from 20,000 × g to 17,000 × g; (v) nucleic acids being eluted to RNA storage solution (Ambion) and storage at -80°C; (vi) random amplification of nucleic acids using the Whole Transcriptome Amplification Kit 2 according to the manufacturer’s instructions (Sigma Aldrich); (vii) amplified products being purified using the Wizard^®^ PCR Cleanup Kit (Promega); and (viii) library preparation using the Nextera XT kit and a repeat purification step. A DNA size selection step was omitted before sequencing as both solid phase reversible immobilisation beads and manual excision from agarose gel did not achieve desired results. Samples were sequenced using the Illumina MiSeq 500 platform (2 × 150bp).

Taxonomic assignment of virome reads was performed using the Vipie platform [56]. Paired-end sequences were uploaded (https://binf.uta.fi/vipie/) using the default parameters with the exception of: (i) read length of 150 base pairs; (ii) subsample of 0.80; (iii) Kmer length of 31; (iv) remapping of hits PER 1,000,000 and (v) minimum total matches of 2. Further details are provided in S2 Methods. The output included a search hit table of all viruses for each sample, which were combined for further analyses.

Functional annotation was performed using the Virsorter pipeline [33] to generate predictions which were searched against the KEGG (34), COG (35) and Pfam [36] databases.

### Inflammatory biomarkers

Calprotectin was extracted and measured as described in a previous study [7] using the PhiCal kit (Calpro, San Diego, CA, US). The lower limit of detection for the assay was 19.5 mg/kg. Calprotectin > 50 mg/kg is considered elevated. Faecal M2-PK was extracted and measured as described in a previous study [11] using the ScheBo Tumour M2-PK kit (ScheBo Biotech, Giessen, Germany). The lower limit of detection of the assay was 1 U/mL.

### Statistical analysis

Statistical analysis was performed in R v3.4.4. Alpha diversity indices (richness and Shannon index) were calculated. For viral taxa, relative abundance was calculated as the proportion of all contigs taxonomically assigned. For functions, relative abundance was calculated as the proportion of all assigned predictions. Bray-Curtis dissimilarities based on relative abundance and presence-absence data were calculated using the vegan package [57] and used to generate non-metric multidimensional scaling (NMDS) plots. Permutational multivariate analysis of variance (PERMANOVA) tests (permutations = 1000) were utilised to test if beta diversity significantly differed among groups (‘CF vs HC’ and ‘male vs female’) and age using the vegan function adonis [57]. A significant difference in abundance of viruses or protein functions between CF and HC groups was assessed using the ANCOM package v1.1–3, which uses non-parametric Kruskal-Wallis tests (Benjamini & Hochberg correction for multiple comparisons, q<0.05) [49]. Continuous variables were compared using a t-test or Wilcoxon signed-rank test for parametric and non-parametric data, respectively (p<0.05 considered statistically significant). Generalised linear models (glm function; using a Gaussian distribution) were constructed to control for age when comparing continuous variables between cohorts. Correlations between continuous variables were assessed using Spearman correlations (Benjamini & Hochberg correction, q<0.05) [58]. Graphs were generated using ggplot2 in R [59].

## Supporting information

S1 Fig Rarefaction curves for CF and HC cohorts. The number of viruses given the number of samples (A & B) and the number of viral sequences per stool sample (C & D).
(TIFF)

S2 Fig (A-E) Relative abundance of all viruses at each taxonomic rank: (A) group; (B) order; (C) family (top 20 most abundant); (D) genus (top 20 most abundant); (E) species (top 20 most abundant). CF and HC subjects ordered in increasing age (from left to right).
(TIF)

S3 Fig Viruses with a significantly different abundance between CF and HC cohorts (at each taxonomic rank) using ANCOM analysis (q<0.05). (A) *Myoviridae* was present in 6/8 (75%) CF and 7/8 (87.5%) HC samples. (B) Unclassified *Gokushovirinae* was present in 4/8 (50%) CF and 8/8 (100%) HC samples. (C) *Faecalibacterium* phage FP Taranis was not present in CF samples (0%), however, was present in 6/8 (75%) HC samples. No statistically significant differences (q<0.05) were identified at the group and order ranks.
(PDF)

S4 Fig COG (A) and Pfam (B) terms with a significantly different abundance between CF and HC cohorts using ANCOM analysis (q<0.05). KEGG (C) terms with a different abundance between CF and HC cohorts which was close to significance (q<0.1). COG3409, peptidoglycan-binding (PGRP) domain of peptidoglycan hydrolases; K08640, zinc D-Ala-D-Ala carboxypeptidase; PF02305.16, capsid protein (F protein).
(PDF)

S5 Fig Scatterplots of the relative abundance of: (A-C) viruses (family, genus, species); (D) a COG terms, (E) a Pfam terms, and (F) a KEGG term, against age in CF and HC cohorts. Cohort mean and 95% confidence intervals are constructed from generalised linear models and presented as lines and shaded regions, respectively.
(TIFF)

S6 Fig Faecal inflammatory biomarkers. Calprotectin (A) was significantly elevated in children with CF compared to HC (98.9 mg/kg (50.1–104.9) vs. 19.5 mg/kg (19.5–30.5), respectively, p = 0.047). M2-PK (B) was significantly elevated in children with CF compared to HC (7.4 U/ml (6.3–22.6) vs. 1.0 U/ml (1.0–1.0), respectively, p = 0.005).
(TIFF)

S1 Table Correlations between the relative abundances of viruses and: (i) anthropometric z-scores, and (ii) inflammatory markers in children with CF. *Spearman correlations (q<0.05) considered statistically significant, with the remaining Spearman correlations (q<0.1) considered close to significance. Analysed virus highlighted in **bold**, and multiple **bold** viruses in a single row indicates consistent results across taxonomic ranks. Calpro, calprotectin.
(DOCX)

S2 Table Correlations between the relative abundances of KEGG, COG and Pfam terms with: (i) anthropometric z-scores, and (ii) inflammatory markers in children with CF. *Spearman correlations (q<0.05) considered statistically significant, with the remaining Spearman correlations (q<0.1) considered close to significance.
(DOCX)

S1 Methods Sample processing for viral metagenomics. (DOCX)

S2 Methods Vipie platform (56) analysis parameters. (DOCX)

S1 Data Meta data: Demographic, clinical and inflammatory biomarker data. (CSV)

S2 Data Sequence characteristics: Read counts, de novo assembly and distribution of all reads. (CSV)

S3 Data Virome table: The abundance of viruses identified in each sample. (CSV)

S4 Data Functional annotation: Results of viral contigs which were searched against KEGG, COG and Pfam databases. (CSV)
