# Supplementary figures and images for: The intestinal virome in children with cystic fibrosis differs from healthy controls
Source: PLoS One. 2020 May 22;15(5):e0233557. doi: 10.1371/journal.pone.0233557 (PMC7244107; doi:10.1371/journal.pone.0233557)

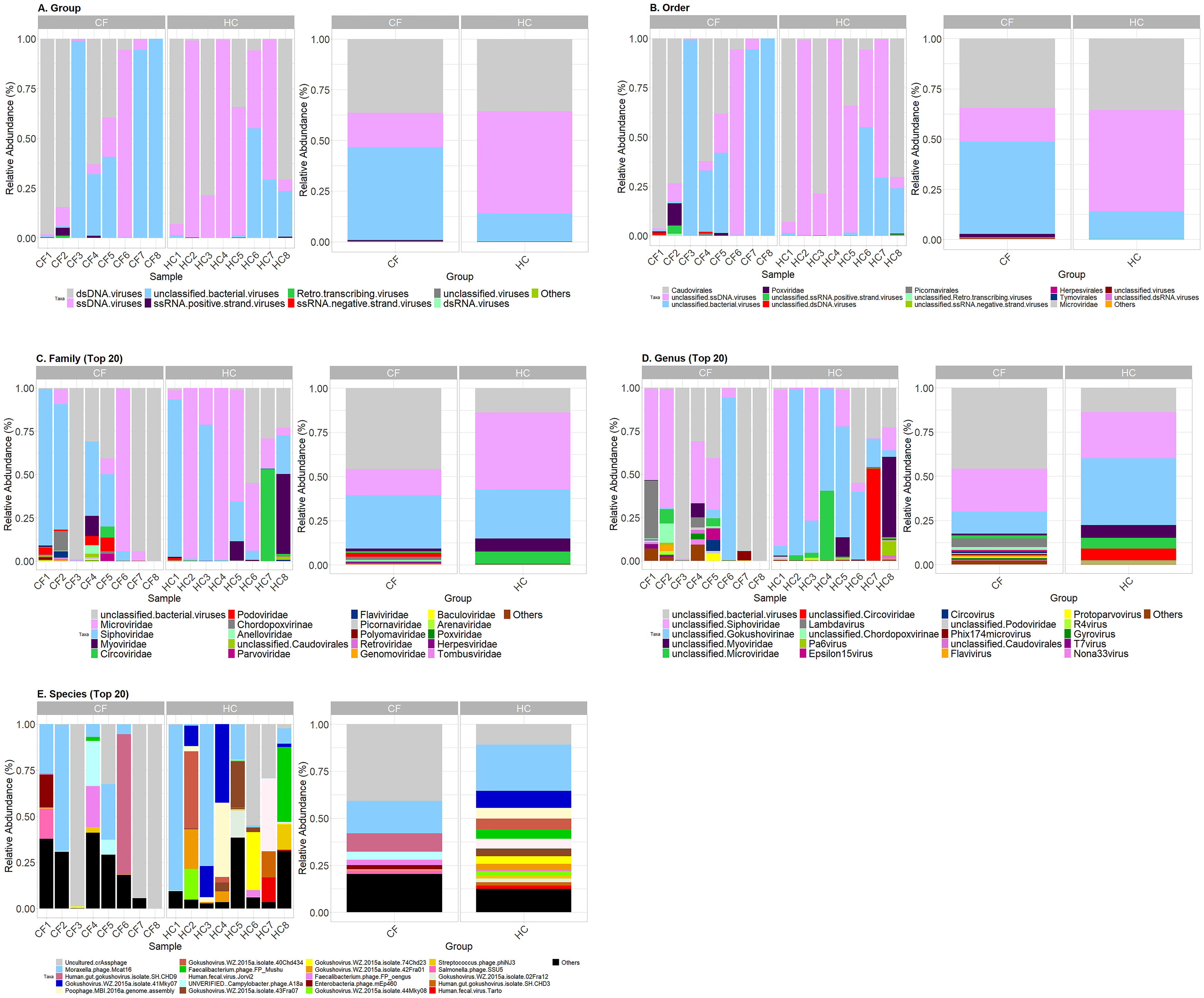

Supplement: S2 Fig — (A-E) Relative abundance of all viruses at each taxonomic rank: (A) group; (B) order; (C) family (top 20 most abundant); (D) genus (top 20 most abundant); (E) species (top 20 most abundant). CF and HC subjects ordered in increasing age (from left to right). (TIF) [file pone.0233557.s002.tif]

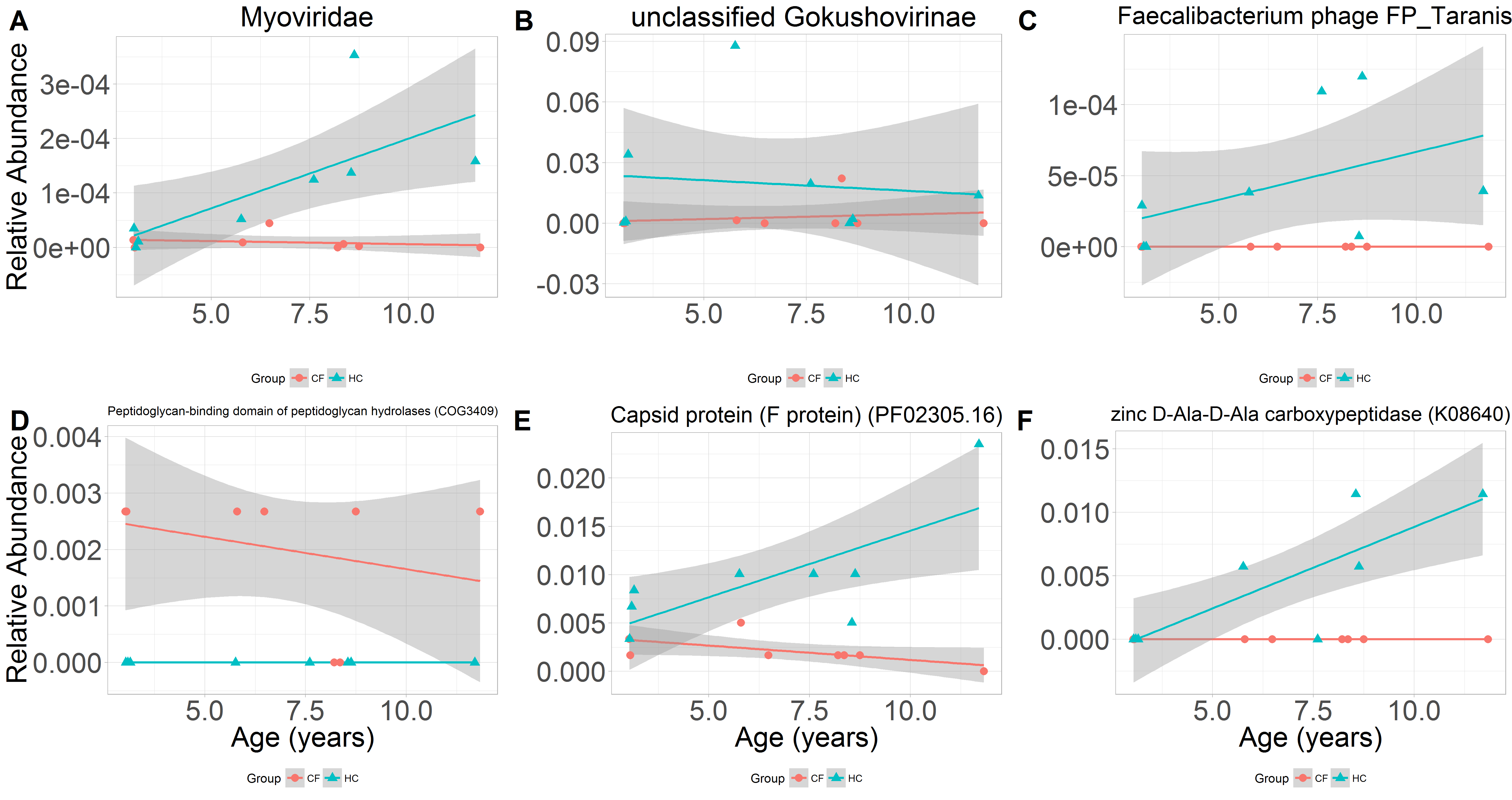

Supplement: S5 Fig — Scatterplots of the relative abundance of: (A-C) viruses (family, genus, species); (D) a COG terms, (E) a Pfam terms, and (F) a KEGG term, against age in CF and HC cohorts. Cohort mean and 95% confidence intervals are constructed from generalised linear models and presented as lines and shaded regions, respectively. (TIFF) [file pone.0233557.s005.tiff]

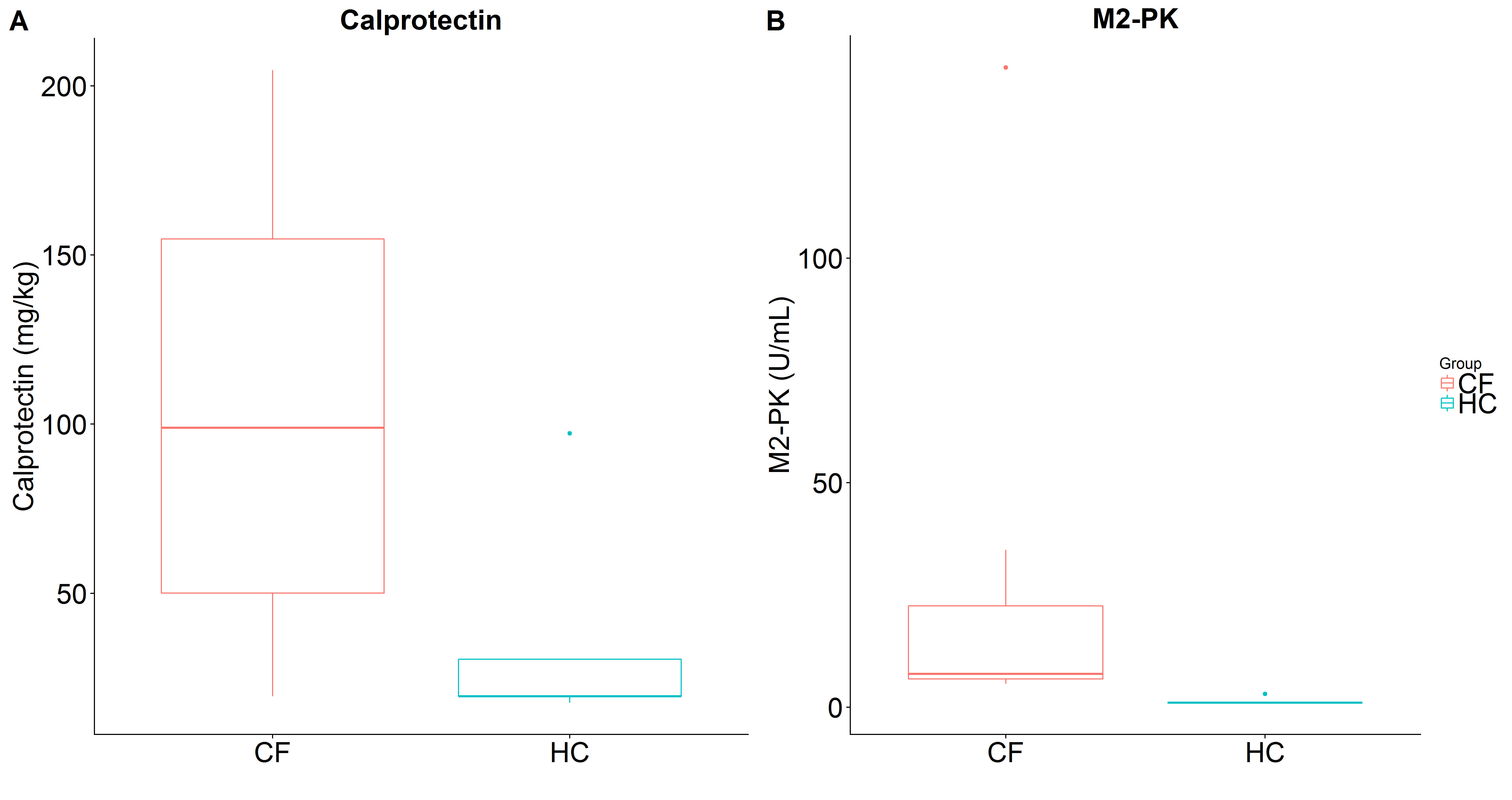

Supplement: S6 Fig — Calprotectin (A) was significantly elevated in children with CF compared to HC (98.9 mg/kg (50.1–104.9) vs. 19.5 mg/kg (19.5–30.5), respectively, p = 0.047). M2-PK (B) was significantly elevated in children with CF compared to HC (7.4 U/ml (6.3–22.6) vs. 1.0 U/ml (1.0–1.0), respectively, p = 0.005). (TIFF) [file pone.0233557.s006.tiff]
