# Supplementary material for: The intestinal virome in children with cystic fibrosis differs from healthy controls
Source: PLoS One. 2020 May 22;15(5):e0233557. doi: 10.1371/journal.pone.0233557 (PMC7244107; doi:10.1371/journal.pone.0233557)
Supplement: S3 Fig — (A) Myoviridae was present in 6/8 (75%) CF and 7/8 (87.5%) HC samples. (B) Unclassified Gokushovirinae was present in 4/8 (50%) CF and 8/8 (100%) HC samples. (C) Faecalibacterium phage FP Taranis was not present in CF samples (0%), however, was present in 6/8 (75%) HC samples. No statistically significant differences (q<0.05) were identified at the group and order ranks. (PDF) [file pone.0233557.s003.pdf]

S3 FIGURE

A. Family

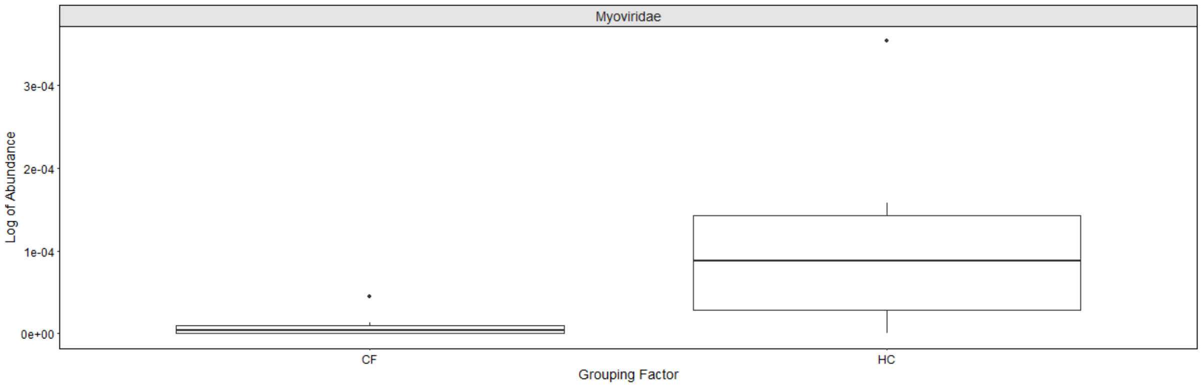

B. Genus

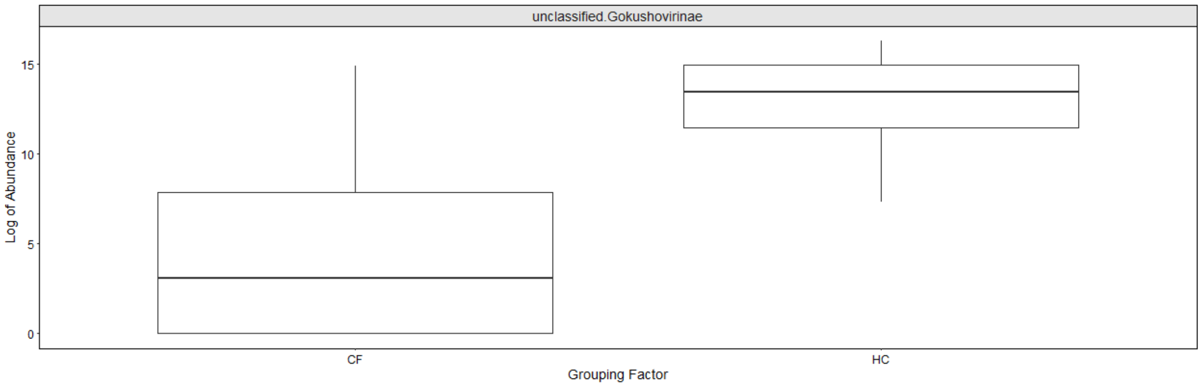

C. Species

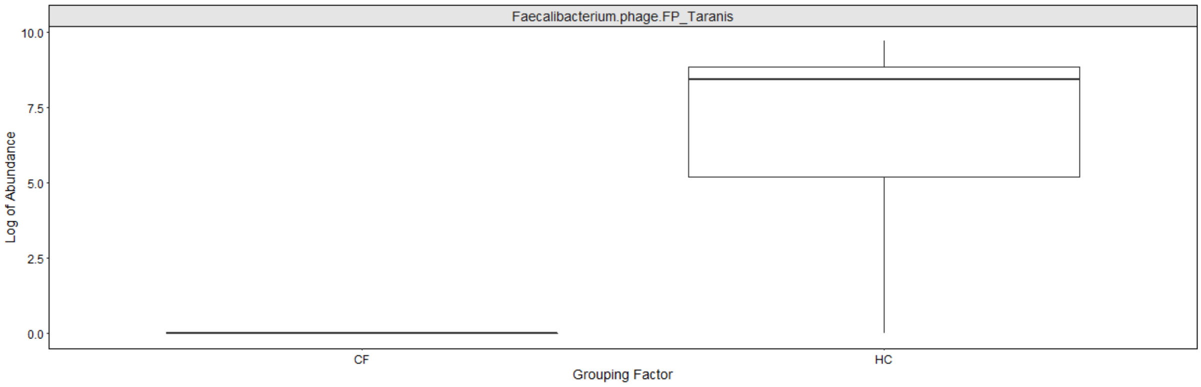

Supplementary Figure 3. Viruses with a significantly different abundance between CF and HC cohorts (at each taxonomic rank) using ANCOM analysis ($q<0.05$). No statistically significant differences ($q<0.05$) were identified at the group and order ranks.
