# Supplementary material for: The intestinal virome in children with cystic fibrosis differs from healthy controls
Source: PLoS One. 2020 May 22;15(5):e0233557. doi: 10.1371/journal.pone.0233557 (PMC7244107; doi:10.1371/journal.pone.0233557)
Supplement: S4 Fig — COG (A) and Pfam (B) terms with a significantly different abundance between CF and HC cohorts using ANCOM analysis (q<0.05). KEGG (C) terms with a different abundance between CF and HC cohorts which was close to significance (q<0.1). COG3409, peptidoglycan-binding (PGRP) domain of peptidoglycan hydrolases; K08640, zinc D-Ala-D-Ala carboxypeptidase; PF02305.16, capsid protein (F protein). (PDF) [file pone.0233557.s004.pdf]

S4 FIGURE

A. COG (q<0.05)

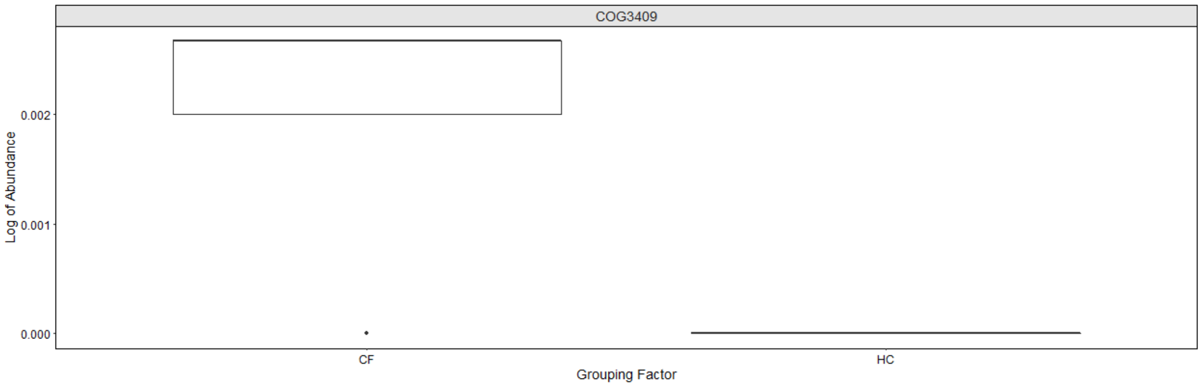

B. Pfam (q<0.05)

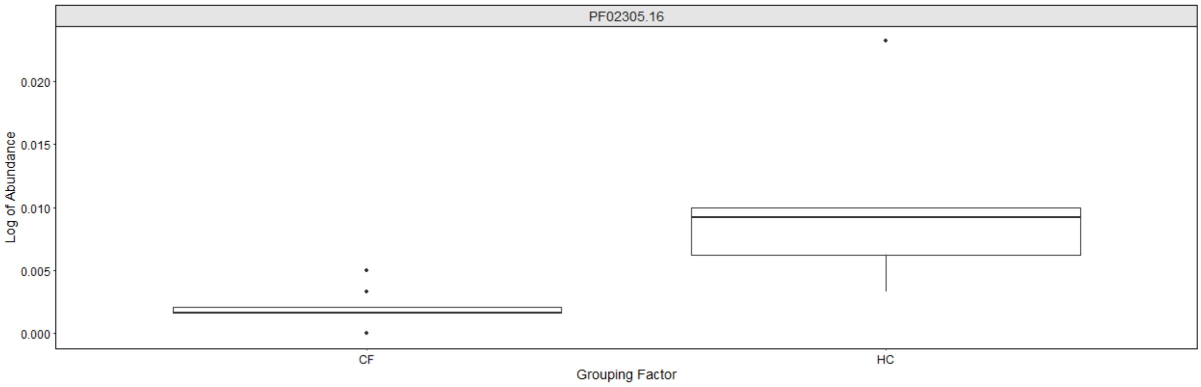

C. KEGG (q<0.1)

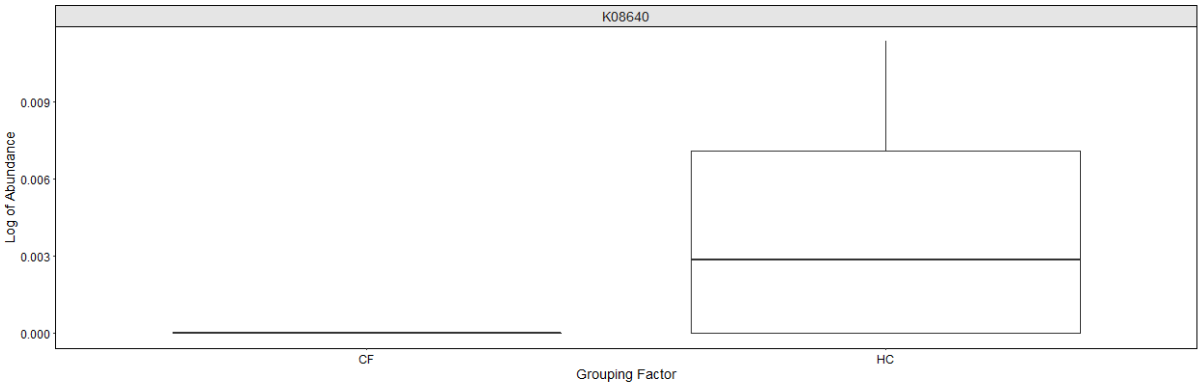

Supplementary Figure 4. COG (A) and Pfam (B) terms with a significantly different abundance between CF and HC cohorts using ANCOM analysis (q<0.05). KEGG (C) terms with a different abundance between CF and HC cohorts which was close to significance (q<0.1). COG3409, peptidoglycan-binding (PGRP) domain of peptidoglycan hydrolases; K08640, zinc D-Ala-D-Ala carboxypeptidase; PF02305.16, capsid protein (F protein).
