# Supplementary material for: The intestinal virome in children with cystic fibrosis differs from healthy controls
Source: PLoS One. 2020 May 22;15(5):e0233557. doi: 10.1371/journal.pone.0233557 (PMC7244107; doi:10.1371/journal.pone.0233557)
Supplement: S1 Table — *Spearman correlations (q<0.05) considered statistically significant, with the remaining Spearman correlations (q<0.1) considered close to significance. Analysed virus highlighted in bold, and multiple bold viruses in a single row indicates consistent results across taxonomic ranks. Calpro, calprotectin. (DOCX) [file pone.0233557.s007.docx]

**S1 TABLE**

| **GROUP** | **ORDER** | **FAMILY** | **GENUS** | **SPECIES** | **Weight** | **Height** | **BMI** | **Calpro** | **M2-PK** |
| --- | --- | --- | --- | --- | --- | --- | --- | --- | --- |
| dsDNA | *Caudovirales* | ***Podoviridae*** |  |  |  |  |  |  | + 0.85* |
| dsDNA | *Caudovirales* | *Podoviridae* | ***T7virus*** |  |  |  |  |  | + 0.80 |
| dsDNA | *Caudovirales* | *Siphoviridae* | *Pa6virus* | ***Propionibacterium phage* PHL092M00** |  |  |  |  | – 0.80 |
| dsDNA | *Caudovirales* | *Siphoviridae* | *Pa6virus* | ***Propionibacterium phage* Ouroboros** |  |  |  |  | – 0.76 |
| dsDNA | *Caudovirales* | *Siphoviridae* | unclassified | ***Bacteroides phage*** **B124-14** |  |  |  |  | – 0.80 |
| dsDNA | *Caudovirales* | *Siphoviridae* | unclassified | ***Klebsiella phage* KPP5665-2** |  |  |  | + 0.73 |  |
| dsDNA | *Caudovirales* | *Myoviridae* | unclassified | ***Enterococcus phage* EFP01** |  | – 0.76 |  | – 0.76 |  |
| **ssDNA** | **unclassified** |  |  |  |  |  |  |  | – 0.86* |
| ssDNA | unclassified | ***Anelloviridae*** |  |  | + 0.79* |  | + 0.79* |  |  |
| ssDNA | unclassified | *Anelloviridae* | ***Gyrovirus*** |  | + 0.71 |  | + 0.71 |  |  |
| ssDNA | unclassified | ***Parvoviridae*** | ***Protoparvovirus*** |  | – 0.87* |  |  |  |  |
| ssDNA | unclassified | ***Microviridae*** |  |  |  |  |  |  | – 0.77 |
| ssDNA | unclassified | *Genomoviridae* | ***Gemycircularvirus*** | ***Chicken stool-associated gemycircularvirus* strain RS/BR/2015** |  |  |  | + 0.76 |  |
| ssRNA + strand | ***Picornavirales*** | ***Picornaviridae*** | ***Enterovirus*** |  | + 0.84* |  | + 0.84* |  |  |
| **ssRNA – strand** | **unclassified** | ***Arenaviridae*** | ***Mammarenavirus*** | ***Lassa virus* strain Soromba-R segment S** |  |  |  | + 0.76 |  |

**Supplementary Table 1.** Correlations between the relative abundances of viruses and: (i) anthropometric z-scores, and (ii) inflammatory markers in children with CF. *Spearman correlations (q<0.05) considered statistically significant, with the remaining Spearman correlations (q<0.1) considered close to significance. Analysed virus highlighted in **bold**, and multiple **bold** viruses in a single row indicates consistent results across taxonomic ranks. Calpro, calprotectin.
