# Supplementary material for: The intestinal virome in children with cystic fibrosis differs from healthy controls
Source: PLoS One. 2020 May 22;15(5):e0233557. doi: 10.1371/journal.pone.0233557 (PMC7244107; doi:10.1371/journal.pone.0233557)
Supplement: S2 Table — *Spearman correlations (q<0.05) considered statistically significant, with the remaining Spearman correlations (q<0.1) considered close to significance. (DOCX) [file pone.0233557.s008.docx]

**S2 TABLE**

|  | **Weight** | **Height** | **BMI** | **Calpro** | **M2-PK** |
| --- | --- | --- | --- | --- | --- |
| **KEGG** |  |  |  |  |  |
| N-acetylmuramoyl-L-alanine amidase (K01447) |  | + 0.85* |  |  |  |
| DNA polymerase III subunit epsilon (K02342) |  |  |  |  | – 0.79 |
| Integrase/recombinase XerD (K04763) |  |  |  |  | + 0.79 |
| Phage terminase small subunit (K07474) |  |  |  | + 0.76 |  |
| Trimeric autotransporter adhesin (K21449) |  |  |  |  | + 0.79 |
| **COG** |  |  |  |  |  |
| Endonuclease III (COG0177) |  | + 0.76 |  |  |  |
| DNA polymerase III subunit epsilon (COG0847) |  |  |  |  | – 0.79 |
| Chromosome segregation ATPase (COG1196) |  |  | + 0.76 |  |  |
| DNA repair photolyase (COG1533) |  | + 0.76 |  |  |  |
| Phage terminase large subunit (COG1783) |  |  |  |  | + 0.79 |
| N-acetylmuramoyl-L-alanine amidase (COG3023) |  | + 0.76 |  |  |  |
| Phage terminase, small subunit (COG3728) |  |  |  | + 0.76 |  |
| Predicted phage phi-C31 gp36 major capsid-like protein (COG4653) |  |  |  |  | + 0.78 |
| Site-specific recombinase XerD (COG4974) |  |  |  |  | + 0.79 |
| Phage-related tail protein (COG5283) |  |  |  | + 0.76 |  |
| **Pfam** |  |  |  |  |  |
| Phage integrase family (PF00589.21) |  |  |  |  | + 0.80 |
| Exonuclease (PF00929.23) |  |  |  |  | – 0.87 |
| Cysteine protease Prp (PF04327.11) |  |  |  | + 0.76 |  |
| Phage tail tube protein (PF06199.10) |  |  |  | + 0.76 |  |
| YopX protein (PF09643.9) |  |  |  | + 0.76 |  |
| Protein of unknown function (PF09674.9) |  | + 0.76 |  |  |  |
| Chlamydia-phage Chp2 scaffold (PF09675.9) |  |  |  |  | – 0.79 |
| AAA proteins (PF13476.5) |  |  |  |  | – 0.87 |
| Domain of unknown function (PF14265.5) |  |  |  | + 0.76 |  |

**Supplementary Table 2**. Correlations between the relative abundances of KEGG, COG and Pfam terms with: (i) anthropometric z-scores, and (ii) inflammatory markers in children with CF. *Spearman correlations (q<0.05) considered statistically significant, with the remaining Spearman correlations (q<0.1) considered close to significance.
