# Supplementary material for: The intestinal virome in children with cystic fibrosis differs from healthy controls
Source: PLoS One. 2020 May 22;15(5):e0233557. doi: 10.1371/journal.pone.0233557 (PMC7244107; doi:10.1371/journal.pone.0233557)
Supplement: S1 Methods — (DOCX) [file pone.0233557.s009.docx]

**S1 METHODS. Sample processing for viral metagenomics.**

The methods used are based on an adjusted protocol of the NetoVIR (Novel Enrichment Technique Of VIRomes) approach to sample preparation for viral metagenomics (1). On overview of the methods is provided in the flow chart below.


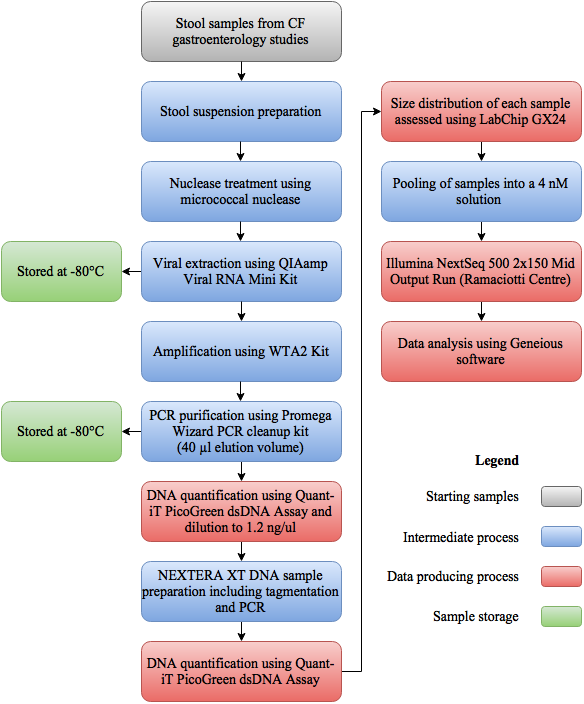


1. Sample collection
   1. A single stool sample from each subject was collected and stored immediately at –80°C, or stored at –20°C (home freezer) until transport to the laboratory for storage at –80°C. Thawing of the sample during transport did not occur.
2. Stool suspension preparation
   1. Approximately 50 mg of frozen stool sample was suspended in 500 µl of sterile phosphate-buffered saline (PBS) to obtain a 10% mass/volume suspension. The suspension was homogenised using a BioShake iQ (Q.Instruments) at 1800 rpm and centrifuged at 17 000 *× g* for 4 min. Next, 160 µl of the supernatant was filtered through a 0.8 µm PES membrane filter at 17 000 *× g* for 1 min (Sartorius Stedium Australia).
3. Nuclease treatment
   1. To 130 µl of the filtered supernatant, 7 µl of 20X nuclease buffer and 2 µl of micrococcal nuclease (NEB) was added. The eppendorf tubes gently inverted 3 times to mix and incubated at 37°C for 2 h. Next, 7 µl of EDTA was added to each tube to stop the reaction.
4. Viral extraction
   1. Viral nucleic acids were extracted using the QIAamp Viral RNA Mini Kit (Qiagen) according to manufacturer’s instruction for the extraction of viral RNA and DNA, with the following amendment: centrifugation speed was lowered from 20 000 x *g* to 17 000 x *g* due to limitations of laboratory equipment. The nucleic acids were eluted in 40 µl of RNA Storage Solution (Ambion) and stored at -80°C.
5. Amplification
   1. Random amplification of nucleic acids was performed using the Whole Transcriptome Amplification Kit 2 (WTA2) (Sigma Aldrich) by adding 0.5 µl of Library Synthesis Solution to 2.8 µl of viral extraction in a 200 µl PCR tube. A tube containing nuclease free water was also included as a negative control. The mixture was mixed by pipetting and placed into a BioRad MyCycler programmed for 95°C for 2 min, then cooled to 18°C. To each tube, 1.7 µl of library synthesis mix consisting of 0.5 µl of Library Synthesis Buffer, 0.78 µl of RNAse free water, and 0.4 µl of Library Synthesis Enzyme was added. The mixture was incubated in the thermal cycler with the cycling conditions shown below:

| **Stage** | **Temperature** | **Time** |
| --- | --- | --- |
| 1 – Hold | 18°C | 10 min |
| 2 – Hold | 25°C | 10 min |
| 3 – Hold | 37°C | 30 min |
| 4 – Hold | 42°C | 10 min |
| 5 – Hold | 70°C | 20 min |
| 6 - Hold | 4°C | ∞ |

1. To the synthesized DNA library, 70 µl of amplification mix was added and incubated in the thermal cycler. The amplification mix contained 60.25 µl of RNAse free water, 7.5 µl of Amplification Mix, 1.5 µl of WTA dNTP mix, and 0.75 µl of Amplification Enzyme per reaction. Thermal cycling conditions as shown below:

| **Stage** | **Temperature** | **Time** |
| --- | --- | --- |
| 1 – Hold | 94°C | 2 min |
| 2 – Cycle (17 cycles) | 94°C | 30 sec |
|  | 70°C | 5 min |

1. PCR Purification
   1. The amplified products were then purified with the Wizard® PCR Cleanup Kit (Promega) according to manufacturer’s instructions. The dsDNA was eluted in 40 µl of nuclease free water and multiple aliquots were stored at -80°C.
2. DNA quantification and dilution
   1. Quantification of dsDNA in the purified products was assessed using the PicoGreen® dsDNA Assay Kit (Life Technologies) and the NanoDrop 1000 Spectrophotometer (Thermo Fisher Scientific) according to manufacturer’s instructions. The fluorescence intensity was measured using a Victor X2 Plate Multilabel Plate Reader (Perkin Elmer) with excitation and emission wavelengths set at 485 nm and 535 nm respectively. Each PicoGreen sample was prepared in duplicate on a Nunc™ F96 Microwell™ Black Polystyrene Plate (Thermo Fisher Scientific) which was read three times. Lambda DNA standards were prepared to range in concentration from 0 to 100 ng/µl. Samples were then diluted using the PicoGreen assay results to a 1.2 ng/µl concentration for Nextera XT library preparation.
3. Library preparation
   1. The Nextera XT library preparation kit was used to prepare samples for sequencing. Tagmentation was done by combining 5 µl of tagment buffer and 2.5 µl of amplicon tagment mix with 2.5 µl of amplified dsDNA. This reaction mix was incubated at 55°C for 4 min and cooled to 10°C. Next, 2.5 µl of neutralise tagment buffer was added to each sample to stop the tagmentation reaction.
   2. A unique combination of 2 index identification sequences was then added to each sample to enable multiplex sequencing. To do this, 2.5 µl of each corresponding index (N7XX and S5XX) and 7.5 µl of Nextera PCR master mix was added to the tagmented DNA. This reaction mix was incubated in the conditions below:

| **Stage** | **Temperature** | **Time** |
| --- | --- | --- |
| 1 – Hold | 72°C | 3 min |
|  | 95°C | 30 sec |
| 2 – Cycle (15 cycles) | 95°C | 10 sec |
|  | 55°C | 30 sec |
|  | 72°C | 45 sec |

- 1. A 3 min tagmentation PCR and various input concentrations were also tested (0.2, 1.2, 1.5, 2.0, 3.0 ng/ µl) in an attempt to reduce the effects of fragmentation and to produce longer DNA fragments

1. PCR purification
   1. Nextera XT library product were purified with the Wizard® PCR Cleanup Kit (Promega) according to manufacturer’s instructions. The product was eluted in 20 µl of nuclease free water.
2. DNA size assessment using electrophoresis
   1. Size of DNA fragments in the samples were assessed using a LabChip GX Touch (Perkin Elmer) according to manufacturer’s instructions.
3. DNA quantification and pooling of samples
   1. Quantification of dsDNA in the purified products was reassessed using the Quant-iT™ PicoGreen® dsDNA Assay Kit (Life Technologies). Samples were pooled at 4 nM concentration according to PicoGreen calculations as per requirements for the Ramaciotti Centre for Genomics, University of New South Wales Sydney.
4. Sequencing
   1. Pooled sample (20 µl) was submitted to the Ramaciotti Centre for a mid-output paired end 2x150 bp sequencing run using an Illumina MiSeq 500 instrument.

**REFERENCES**

1. Conceicao-Neto N, Zeller M, Lefrere H, De Bruyn P, Beller L, Deboutte W, et al. Modular approach to customise sample preparation procedures for viral metagenomics: a reproducible protocol for virome analysis. Scientific reports. 2015;5:16532.
