# Supplementary material for: The intestinal virome in children with cystic fibrosis differs from healthy controls
Source: PLoS One. 2020 May 22;15(5):e0233557. doi: 10.1371/journal.pone.0233557 (PMC7244107; doi:10.1371/journal.pone.0233557)
Supplement: S2 Methods — (DOCX) [file pone.0233557.s010.docx]

**S2 METHODS. Vipie platform (1) analysis parameters.**

1. Quality control
   1. Trim left/right: 10 / 10
   2. Qual cutoff: 10
   3. Insert/read length: 150
   4. MAPQ/Phred: 10
   5. Subsample: 0.80
2. De novo assembly
   1. De novo assembly algorithm: Velvet
   2. Amos: No
   3. Kmer length: 31
   4. Expected coverage/cutoff: auto / 20
   5. Min contig length: auto
3. BLAST parameters
   1. Min percent similar: 80
   2. E value: 0.0001
   3. Number of alignments: 10
4. REMAP/Reduction parameters
   1. Minimum total matches: 2
   2. Remapping of hits PER: 1,000,000
   3. Apply blacklist (vector/synthetic refs): yes
   4. Remove ribosomes (bacteria/others): yes
5. Viral Dark Matter
   1. Allow unmapped viral reads for further analysis: yes

**REFERENCES**

1. Lin J, Kramna L, Autio R, Hyoty H, Nykter M, Cinek O. Vipie: web pipeline for parallel characterization of viral populations from multiple NGS samples. BMC Genomics. 2017;18(1):378.
